# The Role of Follicular Fluid Thiol/Disulphide Homeostasis in Polycystic Ovary Syndrome

**DOI:** 10.4274/balkanmedj.2017.1140

**Published:** 2018-07-24

**Authors:** Esra Nur Tola, Nadiye Köroğlu, Merve Ergin, Hilmi Baha Oral, Abdülkadir Turgut, Özcan Erel

**Affiliations:** 1 Department of Gynecology and Obstetrics, In Vitro Fertilization Unit, Süleyman Demirel University School of Medicine, Isparta, Turkey; 2 Clinic of Gynecology and Obstetrics, University of Health Sciences, Kanuni Sultan Süleyman Training and Research Hospital, İstanbul, Turkey; 3 Clinic of Biochemistry, Gaziantep 25 Aralık State Hospital, Gaziantep, Turkey; 4 Department of Gynecology and Obstetrics, Medeniyet University School of Medicine, İstanbul, Turkey; 5 Department of Biochemistry, Yıldırım Beyazıt University School of Medicine, Ankara, Turkey

**Keywords:** Follicular fluid, in vitro fertilization, polycystic ovary syndrome, thiol/disulphide homeostasis

## Abstract

**Background:**

Oxidative stress has been proposed as a potential trigger in the etiopathogenesis of polycystic ovary syndrome-related infertility. Thiol/disulphide homeostasis, a recently identified oxidative stress marker, is one of the antioxidant mechanism in humans with critical roles in folliculogenesis and ovulation.

**Aims:**

To investigate follicular fluid thiol/disulphide homeostasis in the etiopathogenesis of polycystic ovary syndrome and to determine its association with in vitro fertilization outcome. The study procedures were approved by the local ethics committee.

**Study Design:**

Cross-sectional study.

**Methods:**

Follicular fluid from 22 women with polycystic ovary syndrome and 20 ovulatory controls undergoing in vitro fertilization treatment was sampled. Thiol/disulphide homeostasis was analyzed via a novel spectrophotometric method.

**Results:**

Follicular native thiol levels, as well as the native thiol/total thiol ratio, were lower in the polycystic ovary syndrome group than in the non-polycystic ovary syndrome group (p=0.041 and p<0.0001, respectively). Disulphide levels, disulphide/native thiol, and disulphide/total thiol ratios were increased in the polycystic ovary syndrome group (p<0.0001). A positive correlation between the fertilization rate and native thiol (p=0.01, r=0.53) and total thiol (p=0.01, r=0.052) among polycystic ovary syndrome patients was found. A positive predictive effect of native thiol level on the fertilization rate in the polycystic ovary syndrome group was also found (p=0.03, β=0.45, 95% CI= 0.031-0.643)

**Conclusion:**

Deterioration of thiol/disulphide homeostasis, especially elevated disulphide levels, could be one of the etiopathogenetic mechanisms in polycystic ovary syndrome. Increased native thiol levels are related to the fertilization rate among polycystic ovary syndrome patients and are positive predictors of the fertilization rate among polycystic ovary syndrome patients. Improvement of thiol/disulphide homeostasis could be important in the treatment of polycystic ovary syndrome to increase in vitro fertilization success.

Polycystic ovary syndrome (PCOS) is a common endocrinopathy affecting 4%-12% of women of reproductive age (1). It is associated with heterogeneous clinical manifestations including hyperandrogenemia, insulin resistance, and chronic anovulation, resulting in oligomenorrhea and infertility (2). Impaired folliculogenesis and anovulation are the foci in the etiopathogenesis of PCOS.

Reactive oxygen species (ROS), free radical and non-free radical oxygenated molecules, are produced in many physiological processes and can be beneficial at low concentrations (3). Excessive ROS accumulation can lead to damage to cells, proteins, DNA, and lipids; therefore, ROS levels are regulated by antioxidant mechanisms in the organism (4). A shift in this balance in the direction of ROS is called oxidative stress (OS). ROS, which are generated in the follicle, have essential roles in folliculogenesis and ovulation (5). Granulosa cells produce antioxidants to protect oocytes from the detrimental effects of excessive ROS (6). OS has been cited as an etiopathogenetic factor in female infertility.

The number of studies examining the role of OS in the etiopathogenesis of PCOS has increased in recent years, and it has been suggested that increased OS and/or reduction of antioxidant defense mechanisms may contribute to disease processes in PCOS and its associated conditions, such as infertility and insulin resistance (7,8). One of the defense mechanism against OS in humans is the thiol redox reaction. Thiol consists of a sulfhydryl group, and under conditions of OS these functional groups form reversible disulphide bridges (9). These bonds generate functional and structural changes in proteins (10). Disulphide bonds are reduced to thiol groups via antioxidant mechanisms, and thiol/disulphide homeostasis is maintained in this way (11). Therefore, disulphide bridges are thought to be a marker of OS, whereas thiols are suggested as members of the antioxidant system. Only one side of this balance was measured by Ellman (1979), whereas both sides of thiol/disulphide balance can be measured by the Erel and Neselioglu (11) assay method, allowing the complete evaluation of thiol/disulphide status. Dynamic thiol/disulphide homeostasis, a recently defined OS marker, is considered to have critical roles in various vital processes such as folliculogenesis and ovulation, as well as pathological processes including diabetes, PCOS, and coronary artery disease (4,12). Ovulation is regarded as a brief, inflammatory-like process, and physiological levels of ROS are considered important inducers of ovulation (13). PCOS is a common and well-known cause of anovulatory infertility. Although its exact pathophysiology is not still determined, OS is suggested as a potential precipitating factor in the etiopathogenesis of PCOS (14).

This study aims to analyze the potential pathogenetic mechanism of infertility and anovulation caused by PCOS. Therefore, we sought to assess thiol/disulphide homeostasis, which plays essential roles in fertility, in the developing and preovulatory follicles obtained from PCOS patients undergoing in vitro fertilization (IVF) treatment with a novel, inexpensive, available, and fully automated method (11) and to investigate the association of thiol/disulphide homeostasis with impaired ovulation and fertility. We further evaluated its potential role in predicting IVF outcome among PCOS patients.

## MATERIALS AND METHODS

This cross-sectional trial was carried out in a tertiary IVF center between October and December 2016. Written informed consent was obtained from all participants. The study procedures were approved with the protocol number 72867572-050-3986 by the local ethics committee.

Aiming for a difference of approximately 5 μmol/L in disulphide levels between PCOS and non-PCOS patients, we calculated the sample size of each group with an alpha level of 5% and a β error level of 50%. According to this, a very small number of patients were required (2 patients per group).

Twenty-two women with PCOS (PCOS group), diagnosed by the Rotterdam criteria, with a mean age of 28.5±3.6 years were recruited consecutively from the IVF unit. Twenty additional age-matched women without PCOS (non-PCOS group) undergoing IVF were recruited as the control group. The control group was comprised of women with regular menstrual cycles and normal ovaries on ultrasound examination and no clinical or biochemical profiles of hyperandrogenism. The control group received IVF for tubal factors and included only those women who had salpingectomy for ectopic pregnancy or proximal tubal obstruction because of low-grade infection or fimbrial occlusion with or without mild peritubal adhesions. Tubal infertility associated with hydrosalpinx, severe pelvic adhesions, endometriosis, or pelvic inflammatory disease were excluded.

The exclusion criteria for all participants were secondary causes of hyperandrogenism, endocrinopathy such as hypo/hyperthyroidism or abnormal prolactin levels, any chronic disease or medication use, history of any surgical procedure on the ovaries and uterus, smoking, and alcohol consumption. All participants had normozoospermic male partners.

### Follicular fluid sampling

Patients were prepared for follicle sampling using a standard controlled ovarian stimulation protocol including down regulation of the pituitary gland with a gonadotrophin-releasing hormone (GnRH) antagonist. For ovarian stimulation, recombinant and/or urinary follicle stimulating hormone were used, and doses were adjusted for age, ovarian grade, and body mass index (BMI). Gonadotropin treatment was initiated on day 2 of the cycle and monitored by transvaginal ultrasonography (TV-USG) and serum estradiol levels. When the follicle was 13 mm in diameter, patients received 0.25 mg/day cetrorelix-a GnRH antagonist subcutaneously. When at least three follicles had reached 17 mm in diameter assessed by TV-USG, 250 μg human chorionic gonadotrophin (hCG) was administrated, and oocyte retrieval was performed 36 h after the administration of hCG. Follicular fluid aspirates were centrifuged at 3000 rpm for 10 minutes to pellet follicular cells. Only blood-free samples were stored at -80 °C until enzyme-linked immunosorbent assay. The stage of folliculogenesis was determined by light microscopy. On the same day, intracytoplasmic sperm injection was performed on metaphase II (MII) oocytes, and vaginal progesterone (PG) was administered for luteal support. Oocytes were considered successfully fertilized when two pronuclei were observed 1 day later. The fertilization rate (FR) was accepted as the ratio of fertilized oocytes to MII oocytes. A fresh single embryo was transferred when the embryos had reached at least the 8-cell stage. Implantation was determined by serum hCG levels 14 days after embryo transfer. Clinical pregnancy was defined as the presence of a gestational sac as determined by TV-USG.

### Measurement of thiol/disulphide in follicular fluid

Measurements of thiol/disulphide parameters were performed using an automated spectrophotometric method described by Erel and Neselioglu (11). Native and total thiols were detected simultaneously. First, native thiol levels were measured by a modification of the Ellman method (using the classic Ellman reagent modified by using formaldehyde solution). Dynamic disulphide bridges were reduced to functional thiol groups by sodium borohydride. The unused residual sodium borohydride was depleted by formaldehyde. Thus, total thiol concentrations were determined correctly. The number of disulphide bonds is given by half the value of the difference between total and native thiols. Thiol-disulphide-related ratios were calculated after the measurements were completed.

In the novel method, artifactual oxidation during sample manipulation is avoided by using formaldehyde solution and thus, an accurate measurement of disulfides is frequently achieved. Moreover, there was no gas bubble formation, and thus no light scattering and no light transmission instability were observed in these spectrophotometric measurements. On the other hand, it was shown that the use of formaldehyde did not interfere with the measurements.

### Statistical analysis

Data were analyzed by using SPSS version 22.0 for Windows (SPSS Inc, Chicago, IL, USA). Statistical significance was defined as p<0.05. Continuous variables were tested for normality by using the Kolmogorov–Smirnov test. Mean with standard deviation or median (minimum-maximum) were used as descriptive statistics. Differences between groups were assessed by using the independent-samples t-test and Mann-Whitney U test for parametric and nonparametric groups, respectively. The optimal cut-off points of parameters of thiol/disulphide homeostasis were evaluated by receiver operating characteristic analyses. In order to determine whether thiol/disulphide homeostasis had any predictive effect on FR, implantation, and clinical pregnancy, linear and binary logistic regression analysis were used. The association between thiol/disulphide homeostasis and MII, metaphase I (MI), germinal vesicle (GV), and embryo count were evaluated by Pearson or Spearman correlation analysis.

## RESULTS

### Demographic characteristics and thiol/disulphide homeostasis between PCOS and non-PCOS groups

A total of 42 patients undergoing IVF were divided into two groups. The PCOS group (n=22) consisted of patients diagnosed with PCOS according to the Rotterdam criteria, and the non-PCOS group (n=20) consisted of patients with regular ovulatory cycles. There were no statically significant differences between the two groups according to demographic features such as age, partner’s age, BMI, duration of infertility, or hormone levels at day 3 of the cycle. The total number of retrieved oocytes was significantly higher in the PCOS group (16.5±7.1) than the non-PCOS group (10.7±5.7, p=0.008), as well as the GV number (2.8±3.2 in PCOS and 1.1±0.9 in non-PCOS, p=0.008). However, the MI, MII, and embryo counts were distributed homogenously between the two groups. Demographic characteristics and oocyte retrieval parameters between the PCOS and non-PCOS group are demonstrated in Table 1.

Follicular native thiol levels were decreased in the PCOS group (297.1±37.3 μmol/L) compared with the non-PCOS group (334.2±72.5 μmol/L; p=0.041), as well as the native thiol/total thiol ratio (91.8%±1.9% in the PCOS group, 95%±2.3% in the non-PCOS group; p<0.0001). Disulphide levels and the ratios of disulphide/native thiols and disulphide/total thiols in follicular fluid were higher in women with PCOS (13.2±2.7 μmol/L, 4.5%±1.2%, 4.1%±1.0%, respectively; p<0.0001) compared with non-PCOS controls (8.5±3.2 μmol/L, 2.8%±1.2%, 2.5%±1.2%, respectively; p<0.0001). However, only significantly elevated disulphide levels persisted in the PCOS group in multivariate regression analysis (p<0.0001, OR=2.02, 95% CI=1.30-3.13). Parameters of thiol/disulphide homeostasis in follicular fluid between groups are demonstrated in Table 2.

### Effect of thiol/disulphide homeostasis on IVF outcome in the PCOS group

We investigated whether there was a correlation between the parameters of thiol/disulphide homeostasis and oocyte retrieval parameters, embryo count, and FR. None of the oocyte retrieval parameters were correlated with the parameters of thiol/disulphide homeostasis (p>0.5). The FR was positively correlated with native thiols (p=0.01, r=0.53) and total thiols (p=0.01, r=0.052). Embryo count also was not correlated with the parameters of thiol/disulphide homeostasis (p>0.05).

We also evaluated the predictive effect of thiol/disulphide homeostasis on IVF outcome such as FR, implantation, and clinical pregnancy via regression analysis in the PCOS group. When disulphides and the ratios of disulphides/native thiols, disulphides/total thiols, and native/total thiols were taken as covariates in regression analysis, we found a significant predictive effect of native thiols on FR in the PCOS group (p=0.03, β=0.45, 95% CI=0.031-0.643). However, there was no predictive effect of thiol/disulphide homeostasis on implantation and clinical pregnancy (p>0.05).

## DISCUSSION

In the present cross-sectional study, we demonstrated significant differences in antioxidant and oxidant levels of follicular fluid between women with and without PCOS undergoing IVF via measurement of thiol/disulphide levels. To the best of our knowledge, this is the first study to investigate thiol/disulphide homeostasis in the follicular fluid of women with PCOS. Thiol/disulphide detection is a novel, inexpensive, and fully automated method, which was developed by Erel and Neselioglu (11). In our study, disulphide levels and the disulphide/native thiol and disulphide/total thiol ratios that reflect the OS were found to be higher in the PCOS group than in the non-PCOS group, and native thiol levels and the native thiol/total thiol ratio, the markers of the antioxidant system, were observed to be decreased in the PCOS group. These findings lead us to consider that the balance of thiol/disulphide has shifted toward OS in the PCOS group. Consistent with the results of the present study, significantly elevated levels of malondialdehyde (MDA), superoxide dismutase (SOD), and catalase; decreased levels of thiol; and a negative correlation between serum MDA and thiol levels have been demonstrated in PCOS patients (7). Both elevated OS and antioxidant enzymes (14) and the amelioration effect of antioxidant supplements against OS in PCOS have been demonstrated (15). Ovarian dysfunction causing decreased apoptosis at an early stage of folliculogenesis, which results in the development of multiple follicles, has been blamed in the etiopathogenesis of PCOS (16). Thiols play essential roles in cell proliferation, division, and apoptosis (12). High oxidant levels in PCOS may alter the proliferation and apoptosis of granulosa and theca cells, resulting in multiple unregressed follicles and anovulation (12). Elevated OS markers, defined as ROS and lipid peroxidation, have been found in the follicular fluid of PCOS compared with tubal infertile patients in a study similar to ours (17). In contrast, Yildirim et al. (12) demonstrated elevated serum thiols and decreased disulphide levels in the PCOS group relative to non-PCOS controls; however, the material used in their investigation was the serum, and this discrepancy in the type of sample analyzed could have caused the discrepancy in the results.

We could not found any correlation between total oocytes retrieved, MII, MI, GV, embryo count, and the parameters of thiol/disulphide homeostasis among women with PCOS. The presence of antioxidant enzyme transcripts (copper-SOD and zinc-SOD) during the GV and MII stage in mouse and human and glutathione peroxidase and manganese-SOD during the MII stage indicates their essential role in oocyte maturation (18). Consistent with our results, a critical role of antioxidants in the maturation, ovulation, and fertilization of the oocyte has been demonstrated (19). A positive correlation between low ROS and lipid peroxidation levels and oocyte maturation, defined as the presence of meiotic spindle formation, has been demonstrated in PCOS (17).

We found a positive correlation between FR and native and total thiols. We also determined the predictive effect of thiol/disulphide homeostasis on IVF outcome. We found a predictive effect of native thiol on FR, described as the ratio of MII to fertilized oocytes, whereas we could not find any predictive effect on implantation and clinical pregnancy. An increase in ROS production in granulosa cells is associated with decreased fertilization, poor embryo quality, and a decreased implantation rate (20). A positive relationship of lipid peroxidation and total antioxidant capacity with the pregnancy rate in women undergoing IVF was also reported (5). In a study similar to ours, high follicular fluid ROS levels were found to be associated with low FR both in women with PCOS and those with tubal infertility (17). According to Oyawoye et al. (21), total antioxidant capacity is associated with higher FR. In our previous study, we found increased glutathione and decreased glutathione peroxidase and MDA levels in the granulosa cells of oocytes that achieved fertilization in women with unexplained infertility (3). Borowiecka et al. demonstrated elevated thiobarbituric acid reactive substances and protein carbonyl groups in pregnancy-negative patients compared with those of pregnant patients undergoing IVF, whereas they could not found any significant differences by using the thiol assay with Ellman’s reagent (22).

In conclusion, thiol/disulphide homeostasis is deteriorated, and disulphide levels are increased in PCOS. Increased follicular fluid thiol levels, especially those of native thiol, are positively related to FR among PCOS patients. Improvement of thiol/disulphide homeostasis could be important in the treatment of PCOS to increase IVF success in PCOS. Further prospective studies in larger cohorts are needed to validate the results of this study.

## Figures and Tables

**Table 1:**
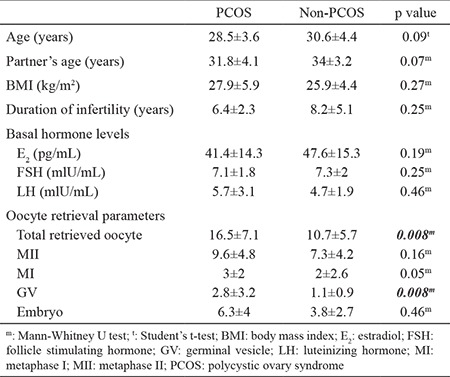
Demographic characteristics and oocyte retrieval parameters between PCOS and non-PCOS groups

**Table 2:**
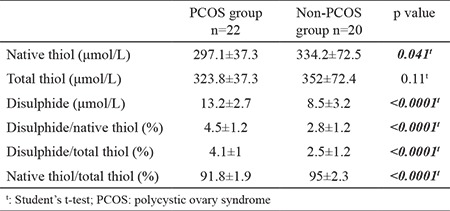
Follicular fluid thiol/disulphide homeostasis between PCOS and non-PCOS group
